# Isolation, screening, and identification of chitinase-producing bacterial strains from riverbank soils at Ambo, Western Ethiopia

**DOI:** 10.1016/j.heliyon.2023.e21643

**Published:** 2023-10-30

**Authors:** Teshome Gudeta Gonfa, Asefa Keneni Negessa, Abebe Olani Bulto

**Affiliations:** a Kotebe University of Education, College of Natural and Computational Sciences, Department of Biology, Addis Ababa, Ethiopia; b Ambo Ubiversity, College of Natural and Computational Sceicnes, Department of Biology, Ambo, Ethiopia; c Animal Health Institute, Sebeta, Ethiopia

**Keywords:** Bacterial strains, Chitin, Chitinases, Clean zone, Riverbank soils

## Abstract

Chitinases are hydrolytic enzymes that dissolve the glycosidic linkages in chitin. Chitin is a cell wall component of fungi and fund in exoskeleten of worms and arthropods. Chitinase has been applied in agriculture, as a biopesticide for the control of plant fungal infections, in medicine, and in waste management. This research aimed to isolate, screen, and identification of chitinase-producing bacteria from riverbank soils. Twenty nine chitinolytic bacteria were isolated from the river bank soil samples, from which 9 of them had strong chitinolytic properties. Chitinase production was determined by zones of hydrolysis produced after 96 h of incubation at 37 °C. The different bacterial isolates were characterized morphologically, microscopically, and biochemically and finally eight strain were identified at species level by Matrix Assisted Laser Desorption Ionization - Time of Flight Mass Spectrometry (MALDI–TOF MS). From the eight, bacterial isolates investigated in this study *Stenotrophomonas maltophilia* showed the highest chitinase enzyme activity (625 μg/mL) followed by *Pseudomonas putida* with the enzyme activity of (553 μg/mL) and the least enzyme activity was recorded for *Lilliottia amnigena* (80 μg/mL). An incubation temperature of 45 °C, neutral pH and an incubation period of 96 h are found to be the optimum condition for the chitinase enzyme production from *Stenotrophomonas maltophilia*. The results of this study indicated the possibility of the production of chitinase from the chitinolytic bacterial isolates, which was highly useful for a variety of applications, including biocontrol of harmful insects and pathogenic fungi as well as in the biochemical, pharmaceutical, and medical sectors.

## 1 Introduction

Chitin is a component of the cell walls of most fungi and some algae and is the second-most abundant polysaccharide. Crabs, lobsters, shrimp, prawns, insects, and other arthropods, as well as crayfish, all have chitin in their exoskeleton material. It is a linear chain of long carbohydrates that consists of N-acetyl-D-glucosamine monomers that are linked by β-1.4 linkage [1]. Chitin is chemically similar to cellulose, except that one glucoside residue has an acetylated or deacetylated amino group in place of one of the hydroxyl groups. Approximately 75 % of the total weight of shellfish, such as shrimp, crabs, and krill, is considered waste, and chitin comprises 20–58 % of the dry weight of this waste [2]. About 10^11^ tons of chitin alone are produced annually in the aquatic biosphere [3]. Due to its numerous biological functions and chemical uses, particularly in the medical field and pharmaceutical industry, chitin has a high economic value.

The chitinase (EC 3.2.11.14) enzyme is capable of hydrolyzing insoluble chitin to its oligomeric and monomeric components and is found in a variety of organisms, including viruses, bacteria, fungi, insects, higher plants, and animals, and plays important physiological roles depending on their origin. Endochitinases and exochitinases are two different categories of chitinases. Endochitinases break down chitin at internal locations to produce GlcNAc multimers. Exochitinases catalyze the hydrolysis of chitin progressively to produce GlcNAc, chitobiose, or chitotriose [4]. There are numerous uses for chitinases, including making single-cell proteins, pharmaceutically significant chitooligosaccharides and N-acetyl D-glucosamine, yeast and fungus protoplast isolation, chitinous waste treatment, pathogenic fungi management, and prevention of malaria transmission. Chito-oligomers, which are made by the enzymatic hydrolysis of chitin, have a variety of uses in industry, agriculture, and medicine, including antibacterial, antifungal, antihypertensive, and food quality enhancement. Furthermore, chitinases play a major role in degrading the chitinous waste from the seafood industry and thus retain the carbon-nitrogen balance in the environment through the utilization of crustacean waste [5]. The need for microbial chitinase production has increased, and it serves two purposes: (i) reduce environmental hazards and (ii) increase the production of industrially important value-added products. There have been reports of bacteria from the genera *Aeromonas*, *Enterobacter*, *Chromobacterium*, *Arthrobacter*, *Flavobacterium*, *Serratia*, *Bacillus*, *Erwinia*, and *Vibrio* degrading chitin in aquatic environments [6]. From whale leftovers, other research has isolated the genera *Eubacterium*, *Streptococcus*, and *Clostridium,* as well as the species of *Bacillus licheniformis* [7]; *Acinetobacter johnsonii* and *Bacillus amyloliquefaciens* from shrimp residues; additional genera, including *Serratia* and *Streptomyces,* from crustacean residues; and *Bacillus licheniformis* from food industry liquid residues [8]. The availability of chitin in the environment is indicated by the presence of chitinolytic microorganisms. However, it is still believed that more effective chitinolytic microorganisms are required for the efficient degradation of chitin. Thus, it is required to search for stronger chitinolytic microorganisms which can break down chitin in materials. To our knowledge, there have been no reports of bacteria degrading chitin from the riverbank soils of Ethiopia. Hence, this study's objective was to isolate, screen, and identify the best chitinolytic bacteria from different riverbank soil samples and to optimize the fermentation conditions for optimum chitinase production.

## 2 Materials and methods

### 2.1 Soil sample collection and processing

The riverbank soil samples were collected from Huluka and Taltale (Ambo town). which is located in the West Shewa Zone of the Oromia Regional state, 116 km (80 miles), west of Addis Ababa, the capital city of Ethiopia. It has a latitude and longitude of 37°47′30″ E− 37°55′15″ E. and an elevation of 2100 m [9]. The riverbank soil samples were collected in clean polyethylene bags and transported to the microbiology laboratory, department of Biology, College of Natural and Computational Sciences, of Kotobe Metropolitian University and the soil samples were serially diluted from 10^−1^ to 10 ^−7^ [10]^.^

### 2.2 Media preparation, inoculation, and incubation

The selective media for isolation of chitinolytic bacteria was prepared following [11], containing g/L: colloidal chitin 1 %(W/V): Na_2_HPO_4_, 6.0, K_2_HPO_4_, 1, NH_4_Cl, 0.5, KH_2_PO_4_ 3, MgSO_4_.7H_2_O 0.12, NaCl, 0.5, yeast extract 0.05, agar 15 and at (pH 7) and autoclaved at 121 °C for 15 min and amended with Fluconazole at a concentration of 250 mg/mL to minimize fungal contamination [12]. The sterilized medium was poured into sterilized plates and cooled to room temperature and inoculated with 100 μL of the 10^−4^ to 10^−7^ dilutions of soil suspension by pour plating techniques and incubated at 37 °C for 4 days [13]. The chitin-degrading bacteria were selected based on clean diameter after 96 h of incubation and further screening was done for maximum enzyme activity in chitin broth media [12].

### 2.3 Colloidal chitin preparation

Chitin flex was purchased from USA online shopping from Amazon.com and 1 g of it was taken in mortar and pestle, added 5 mL of acetone, and ground gently for 10 min. Then 40 mLconcentrated HCl was added to the ground chitin and agitated for 3 h to dissolve the chitin, and then placed at 4 °C overnight. The chitin solution was filtered and the filtrate was made up to 250 mL volume by using 50 % ethanol with constant stirring. The solution was centrifuged at 10,000 rpm for 20 min. The precipitates of chitin obtained were washed with distilled water until neutral pH was obtained. Distilled water was added to form 2 % colloidal chitin and this stock solution was stored at 4 °C until use [14].

### 2.4 Morphological, microscopic, and biochemical characterization of the bacterial isolates

Colony morphology: shape, size, margin, constancy, and color were recorded for fully grown bacterial isolates. Microscopic characterization: gram staining, cell size, cell shape, and arrangements, were recorded for the bacterial isolates. Biochemical characterization: the ability to hydrolysis starch, utilization of Triple Suger Irion, Citrate utilization, Indole production, H_2_S production, Motility, Oxidase test, Gelatin hydrolysis, Catalase test, Methyl red test, Vegas Proskauer test, were performed [15].

### 2.5 Identification of bacterial isolates by Matrix Assisted Laser Desorption Ionization - Time of Flight Mass Spectrometry (MALDI–TOF-MS)

Isolates were identified by MALDI–TOF-MS (Bruker Daltonics, Bremen, Germany) using a formic acid-based direct, on-plate preparation method [16]. In this method, the colonies were spread on the plate and 1 μL of 70 % formic acid per well was deposited onto the MALDI–TOF MS steel anchor plate (BigAnchor 96-well plate; Bruker Daltonics) and allowed to dried. 1 μL of Batcterial Testes Standard (BTS) was deposited onto each of the assigned BTS QC positions and allowed to dry. The dried mixture was overlain with 1 μL of matrix solution (α-cyano-4-hydroxycinnamic acid (HCCA); Bruker Daltonics), dissolved in 50 % acetonitrile, 47.5 % water, and 2.5 % trifluoroacetic acid and allowed to dry before analysis using a MALDI Biotyper. A MicroFlex LT mass spectrometer (Bruker Daltonics) was used for the analysis. The spectra were analyzed using Bruker Biotyper 3.0 software. The manufacturer-recommended cutoff scores were used for identification, with scores of ≥2.000 indicating identification to the species level, scores between 1.700 and 1.999 indicating identification to the genus level, and scores of <1.700 indicating no identification. The isolates producing scores of <1.700 were retested once, and the highest score was used for the final analysis.

### 2.6 Chitinase enzyme assay

Chitinolytic activity was estimated by 3,5 dinitrosalicylic acid (DNS) method using colloidal chitin as a substrate according to the method described by Divarta [12] with some modification. Enzyme solution (1.0 mL) was allowed to react with 1.0 mL of 0.5 % colloidal chitin in (1 mL) of 0.1 M citrate buffer (pH 7.0) for 30 min, the mixture was incubated at 37 °C in a shaking water bath. After incubation, the reaction was stopped by adding 2 mL of DNS reagent and heating it for 10 min in a boiling water bath. After cooling, the colored solution was centrifuged at room temperature for 10 min at a speed of 10,000 rpm and the supernatant's absorbance was measured at 540 nm in comparison to the control. One unit of chitinase activity was defined as the amount of enzyme that liberates of reducing sugar per min per mL.

### 2.7 Determination of chitinase enzyme activity from the different bacterial isolates

The different Growth parameter and their influences on enzyme activities were determined as follows. For the determination of optimum temperature, the temperature range of 25 °C, 30 °C, 37 °C, 45 °C, 50 °C, and 55 °C were evaluated after five days of incubation. For the determination of the optimum pH of the medium; pH values (4, 5, 6, 7, 8, and 9) were evaluated. The pH was adjusted using HCl and NaOH. For the optimum incubation time, the incubation period from 24hrs to 120hrs was evaluated by taking samples every 24hrs intervals. For the evaluation of all of the above parameters media containing 1 % colloidal chitin was prepared, and the broth 100 mL was taken in each flask. Bacterial inoculum was prepared in 25 mLof the same medium in a 100 mL flask. Suspension of the culture was prepared to have 0.8 O.D at 600 nm and 100 μL of inoculum was added in each flask. Flasks were adjusted or incubated in specified conditions and the activity of the enzyme produced was measured by using a spectrophotometer at 540 nm of wavelength [13].

### 2.8 Glucose standard curve

Glucose standard curve was used to determine the amount of reducing sugar in chitinase enzyme produced by chitinases bacterial isolate. The glucose solution was prepared by adding 100 mg glucose powder in to 100 mL distilled water [17]. This solution was serially diluted in different test tubes by adding distilled water. In the different test tubes, different concentrations of glucose solutions were added. To each concentration of 2 mL of glucose in a test tube 2 mL of DNS solution was added. Then, the test tubes were boiled at 100 °C for 10 min in a water bath and cooled in tap water and 6 mL of distilled were added. The intensity of color of each solution of optical density (OD) was measured by spectrophotometer at 540. A graph was plotted with the acquired data where x-axis was labeled with glucose concentration (mg/mL) and y-axis was labeled absorbance 540 nm.

### 2.9 Data analysis

All the quantitative data collected were entered into the data editor view of SPSS for statistical analysis. And analyzed by One-Way Analysis of Variance (ANOVA) and pair wise comparison was done by Tukey test.

## 3 Results and discussion

### 3.1 Isolation of chitinase producing bacterial strains

The riverbank soils were collected from Ambo, western Ethiopia for the isolation of chitin-degrading bacteria and 29 bacterial isolates were obtained from the different riverbank soils which were given code HS or TS following the number of soil samples I-IV were assigned to each bacterial isolate, and then 9 bacterial isolates showing a clean zone diameter from 20 to 35 mm were selected for further screening and optimization studies. The code of the nine bacterial isolates are: HSI1**,** HSII1, HSII2, HSII3, HSII4, HSIII1, HSIII2, TSI1, and TSIII1. The finding of this study was analogous with the reports in the literature. The clear zone surrounding the colony shows chitinase activity to break down chitin compounds in the medium [18]. Similar observations were reported by Ref. [12], where they indicated “the culture filtrates of 2-day-old chitinolytic bacteria were tested for the presence of chitinase enzyme by well diffusion method [19]. Ajayi et al., isolated thirty-six pure bacteria from the skin and gut of catfish (*Clarias gariepinus)* of which 14 isolates were found to produce a clear zone (>10 mm). The total of 63 morphologically different chitinolytic bacterial colonies from 10 samples of Salted and fermented shrimp were isolated based on colloidal chitin degradation and zone of clearance (>0.2 cm) but, only 2 colonies were selected for secondary screening in broth media [20]. The isolation of 167 colonies of bacteria indicated that had chitinolytic properties obtained from rotten Penaeus monodon shells from which only 17 isolates showed strong enzyme activities [21]. The reported the isolation of 71 bacteria from seafood wastes and wastewater effluent from which, only 32 were found to be chitinase positive [22].

### 3.2 Characterization of chitinase-producing bacteria

**Fig. 1:**
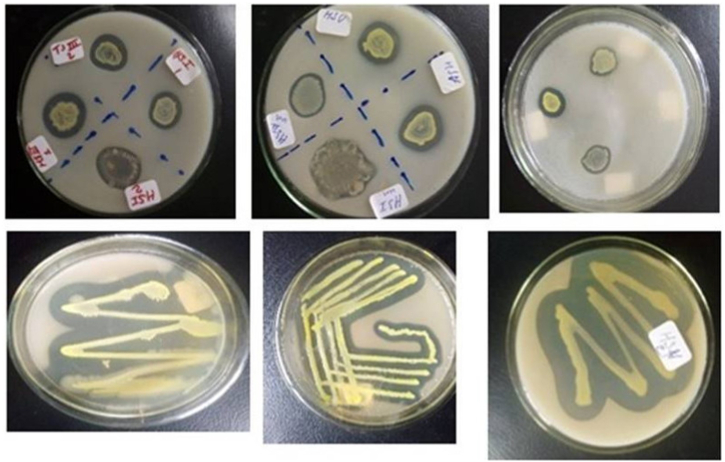
Different colonies of chitinolytic bacterial isolates and the clear zone formed due to chitin degradation.

**Fig. 2:**
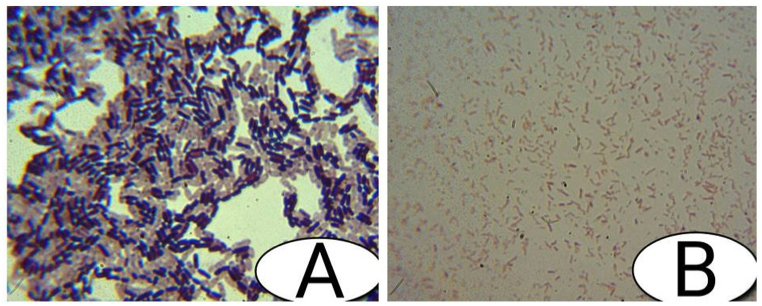
The gram staining micrograph from the light compound microscope of the bacterial isolates HSI1 (A) and TSIII3 (B).

The nine effective chitinolytic bacteria isolated from the riverbank soils were characterized for morphological, microscopic, and biochemical characteristics. And the results of these characterizations are given (Table 1, Table 2, Table 3) and Fig. 1, Fig. 2). The chitinolytic activities of the 9bacterial isolates measured in mm are also shown.

**Table 1:** Morphological Characteristics of the bacterial isolates.

S/N	Isolate code	Colony color	Colony shape	Colony size	Colony texture	Clean zone diameter
1	HSI1	Gray	Irregular	Large	Mucoid	21 mm
2	HSII1	Gray	Circular	Small	Mucoid	28 mm
3	HSII2	Yellow-orange	Circular	Large	Nonmucoid	24 mm
4	HSII3	Creamish gray	Circular	Large	Mucoid	31 mm
5	HSII4	Yellow Light	Circular	Small	Mucoid	25 mm
6	HSIII1	Orange	Circular	Small	Nonmucoid	30.5 mm
7	HSIII2	Orange yellow	Circular	Small	Dry	29 mm
8	TSI1	Yellow	Round	Medium	Mucoid	35 mm
9	TSIII1	Yello Gray	Circular	Small	Nonmucoid	33 mm

**Table 2:** Microscopic characteristics of the bacterial isolates.

S/N	Isolate code	Gram reaction	Cell shape	Cell arrangement	Cell size
1	HSI1	+	Rod	Single	large
2	HSII1	–	Cocci	Single	Large
3	HSII2	–	Rod	Single	small
4	HSII3	–	Rod	Single	Large
5	HSII4	–	Cocci	Chain	Small
6	HSIII1	+	Rod	Single	Small
7	HSIII2	+	Cocci	Single	Small
8	TSI1	–	Rod	Single	Medium
9	TSIII1	–	Rod	Chain	Medium

**Table 3:** Biochemical characteristics for isolates strains.

S/N	Properties	HSI1	HSII1	HSII2	HSII3	HSII4	HSIII1	HSIII2	TSI1	TSIII3
1	Motility test	Motile	Motile	Non motile	Non motile	Non motile	Non motile	Non motile	Motile	Motile
2	Indole	-v	+v	-v	+v	-v	-v	-v	-v	-v
3	Catalase	+v	+v	-v	+v	+v	+v	+v	+v	+v
4	Oxidase	-v	-v	-v	-v	-v	-v	-v	-v	-v
5	Citrate utilization	+v	-v	-v	+v	-v	-v	-v	-v	-v
6	Methyl Red	-v	-v	-v	-v	-v	-v	-v	-v	-v
7	VP	-v	-v	-v	-v	-v	-v	-v	-v	-v
8	Starch hydrolysis	+v	+v	+v	+v	+v	+v	+v	+v	+v
9	Chitin hydrolysis	+v	+v	+v	+v	+v	+v	+v	+v	+v
10	Gelatine hydrolysis	-v	-v	-v	-v	-v	-v	-v	-v	-v
**Triple Irion sugar test**										
11	Glucose	+v	+v	+v	+v	+v	+v	+v	+v	-v
12	Sucrose	+v	+v	+v	+v	+v	+v	+v	+v	+v
13	Lactose	+v	+v	+v	+v	+v	+v	+v	+v	+v
14	Gas production	+v	+v	+v	+v	+v	+v	+v	+v	+v
15	H_2_S	-v	-v	-v	-v	-v	-v	-v	-v	-v

### 3.3 Identification of chitinase-producing bacterial isolates by MALDI-TOF MS

Now a days, a variety of technology advancements for microorganism identification, like Matrix Assisted Laser Desorption Ionization - Time of Flight Mass Spectrometry (MALDI-TOF MS), have been effectively adopted in microbiology laboratories all around the world. For the correct identification of microorganisms, MALDI-TOF-MS is a valuable, quick, accurate, and straightforward technology [23]. The proteomics identification by MALDI-TOF biotype (Matrix Assisted Laser Desorption Ionization - Time of Flight) Mass Spectrometry was used for species identification of the 9 selected chitinolytic bacteria [24]. From the nine bacterial isolates analyzed by MALDI-TOF MS for species-level identification, eight isolates were identified with a score value > 2.0, and only one bacterial isolate was not identified. The names of the several bacterial species that were isolated were listed in the table along with the isolate code score values (Table 4). According to Ref. [25], MALDI-TOF-MS was able to properly identify 88.8 % (128/144) of species and 92.3 % (133/144) of genus-level organisms. 88.9 % of the bacteria examined in this study were accurately identified to the species level by MALDI-TOF-MS. Numerous studies have emphasized the benefits and effectiveness of MALDI-TOF-MS over competing techniques, including its speed, minimal sample volume requirements, and cheap reagent costs. This technology, which is primarily employed for the detection of aerobic bacteria, has been the subject of numerous investigations, and it is now widely used in clinical laboratories all over the world. The identification of *Clostridium* isolates of 108 were successfully identified by MALDI-TOF-MS at the genus and species levels were indicated 96 % of identification rate. Other investigations compared MALDI-TOF-MS and VITEK 2 to find whether approach is more appropriate for identifying anaerobic bacteria, and the majority of these studies came to the conclusion that MALDI-TOF-MS was superior [26]. *Bacillus* was among the bacterial chitinase producers. *Serratia, Chromobacterium, Klebsiella, and Streptomyces* were some of the others. The chitinolytic bacteria were recognized as belonging to the genus *Bacillus*, which was created by the PCR amplification and biochemical test [27]. Similarly, six chitinases from *Bacillus circulans* WL-12 were reported by Ref. [28]. Members of the genus *Bacillus* are well known for their potential to secrete many degradative enzymes such as chitinases [29]. Some of the effectient chitinolytic bacterial species identified in this study were new which not yet reported in the literature this may be due to the fact that the soils samples were collected from the river bank.

**Table 4:** Identified bacterial strains by MALDI-TOF.

S/N	Strains Code	Sample Name	Sample ID	Organism (best match)	Score Value	Nature of organism
**1**	HSI1	C2	25677	*Rhodococcus marinonascens*	2.23	Bacteria
**2**	HSII1	C3	25678	*Aeromonas media*	2.25	Bacteria
**3**	HSII2	C4	25679	*Lilliottia amnigena*	2.27	Bacteria
**4**	HSII3	C5	25680	No peaks found	0	Unkown
**5**	HSII4	C6	25681	*Pseudomonas congelans*	2.16	Bacteria
**6**	HSIII1	C7	25682	*Ligilactobacillus salivarius*	2.15	Bacteria
**7**	HSIII2	C8	25683	*Lactobacillus amylovorus*	2.14	Bacteria
**8**	TSI1	C10	25685	*Stenotrophomonas maltophilia*	2.18	Bacteria
**9**	TSIII1	C11	25686	*Pseudomonas putida*	2.29	Bacteria

### 3.4 Determination of enzyme activities

**Fig. 3:**
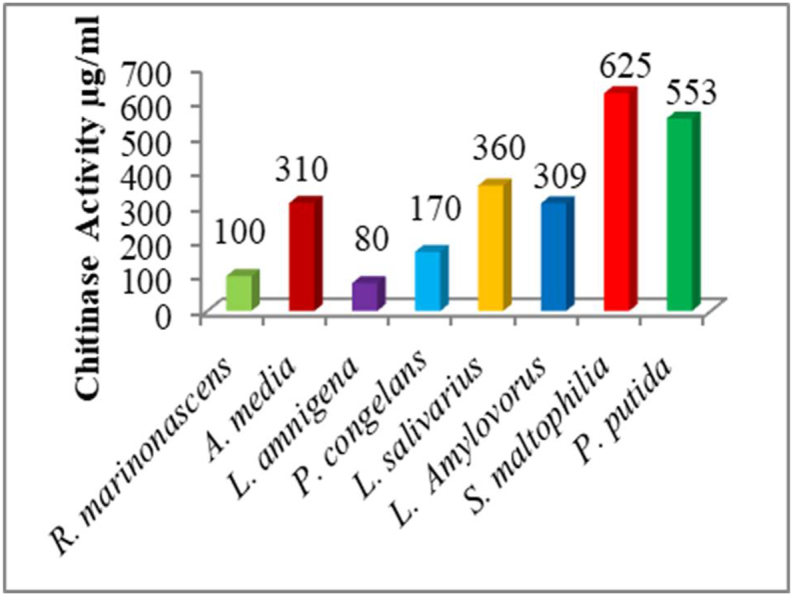
Chitinase activities of the different bacterial isolates.

From the selected potential isolates, the chitinase enzyme activities were confirmed by a specific enzyme assay for chitinase. The Chitinase enzyme activities were done by the DNS method and evaluated from glucose standard curve. From the ten, bacterial isolates *Stenotrophomonas maltophilia* showed the highest enzyme activity (625 μg/mL) followed by *Pseudomonas putida* with the enzyme activity of (553 μg/mL) and the least enzyme activity was recorded for *Lilliottia amnigena* (80 μg/mL) (Fig. 3). The rest of the chitinolytic bacterial isolate showed enzyme activities between the highest and the lowest. Earlier studies have reported the chitinase production by *Alcaligenes faecalis* AU02 to be a maximum (258 U/mL) after 48 h at 37 °C in a medium containing 1 % shrimp and crab shell powder in basal medium (pH 8.0) [30]. The chitinolytic activity in the culture supernatant of *Bacillus pumilus* reached a maximum of 79.8 U/100 mL after 8 days of incubation using a basal medium [31]. In comparison to these findings, our results show an higher chitinase enzyme activities. The chitinase activity of the *Bacillus* sp. *Bacillus* sp. strain SCH-1 increased along with the cell growth and reached a maximum (12.52 unit/mg protein) when the cell growth reached the stationary phase at about 4 d of incubation. Some reporte indicated that, the chitinase activity for the *Paenibacillus* sp. SCH2 strain also increased with the cell growth and reached maximum (5.12 unit/mg protein), when the cell growth reached the death phase at 4 d of incubation [32]. The chitinase isozymes in the chitin medium showed 2 bands at 41 and 50 kDa for the *Bacillus* sp. strain SCH-1 (The chitinolytic index of *P. stuartii* reaches about 4.46 after incubation for 48 h at 37 °C, which is higher compared to other chitinolytic bacteria isolated from other sources [21], such as *Acinetobacter johnsonii* (2.069) and *Bacillus amyloliquefaciens* (2.084), which were isolated from the solid and liquid waste of shrimp shells (after incubation for 7 days at 30 °C) [33]. Bacteria produce several chitinases, to hydrolyze different form of chitin found in nature. Chitins can vary by the arrangement of NAG strands, the degree of deacetylation, and the presence of crosslinked structural components, such as proteins and glucans. Bacterial chitinases belong to family 18 of the glycosyl hydrolases [4].

### 3.5 Effect of incubation temperature on chitinase activity

**Fig. 4:**
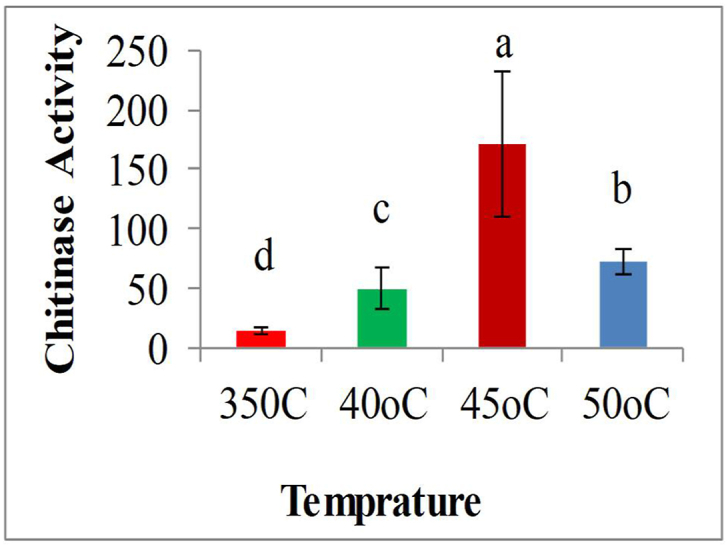
Effects of different incubation Temperature on chitinase enzyme activity.

The different incubation temperatures evaluated significantly affected the chitinase enzyme activities from the different bacterial strains (F_3_ = 340.8; P < 0.0001). The optimum temperature for the highest chitinases enzyme activities was recorded at 45 °C. Bacterial strain(Fig. 4) *Stenotrophomonas maltophilia* (TSI1) showed the highest chitinase activity (625 μg/mL); followed by *Pseudomonas putida* 553 μg/mL while the incubation temperature which stimulated the least chitinase enzymes was found to be 35 °C by bacterial isolate *Rhodococcus marinonascens* (HSI1) showing the chitinase enzyme activity of 24.453 μg/mL. The rest of the bacterial isolates and the different incubation temperatures evaluated showed the intermediate results of higher enzyme activity and that of the lowest enzyme activity (Fig. 4). The results observed in this study were similar to the results reported in the literature *Vibrio alginolyticus and Bacillus* sp at 45oC, [34] respectively. *P. alcaligenes* with the enzyme activity of 47.9 U/ml [35], all showed optimum chitinolytic activity at an incubation temperature of 45 °C., but a slight variation was reported by other reports the optimum temperature for chitinase activity from *Serratia marcescens* at 50 °C [36], *Bacillus pumilus* SG2 55 °C [12]. Temperature is an important environmental factor that affects the growth and metabolite production by microorganisms. In this study, the profile of the influence of*, the* temperature on chitinase production was detectable between 35 °C and 50 °C. The enzyme activity was found to increase with temperature increase up to 45 °C, and then to decrease sharply with further increase in temperature [37]. Optimal chitinase production was reported to be 30 °C in *Streptomyces tendae* [38], 45 °C in *Bacillus licheniformis* SK-1 [39] and 60 °C in *Pseudomonas* sp. [40]. Nawani, N and. Kapadnis, B [41] reported maximum enzyme production after 120 h of incubation with isolate *Streptomyces* sp. NK1057, whereas Wang, J reported [40] maximum enzyme production by *Pseudomonas* sp. at 72 h, and Wang, S and. Hwang, J [2] reported maximum enzyme production by *Bacillus cereus*, *Bacillus alvei* and *B. sphaericus* at 48 h of incubation.

### 3.6 Effect of different pH change on chitinase production

**Fig. 5:**
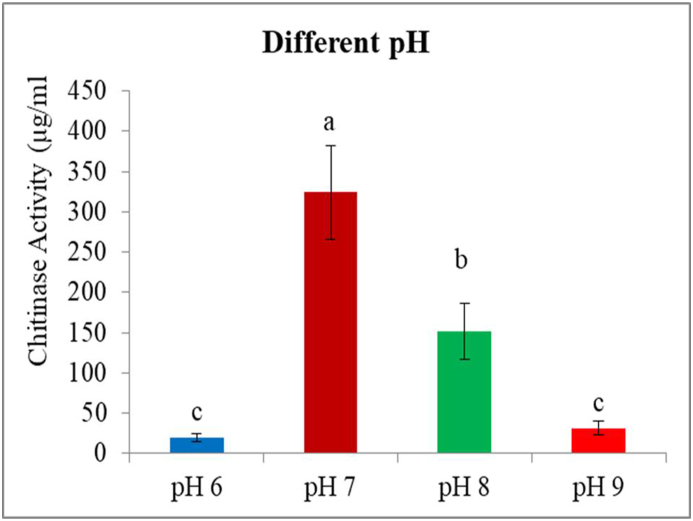
Effects of different pH of the medium on chitinase enzyme activity.

The pH of the medium significantly affected enzyme activity from the different bacterial isolates (F3 = 96.08; P < 0.0001). In this study, the highest chitinase activity was recorded for bacterial strain *Stenotrophomonas maltophilia* (TSI1) 620 μg/mL; followed by *Pseudomonas putida* (TSIII1) 545 μg/mL. The smallest enzyme activity was recorded for the *Lelliottia amnigena* HSIII1 85 μg/mL (Fig. 5). The rest of the PH of the medium and the different bacterial strains showed between the highest and lowest enzyme activities. The majority of the reported results in the literature indicated maximum activity of chitinase activities at neutral or slightly acidic pH, whereas in our study the maximum chitinase activities were obsessed at neutral and basic PH medium. Reports indicated that chitinase activity of *A. hydrophila* HS4 was noted as 93.27 U/mL at pH 8 and for *A. punctata* HS6 as 73.43 U/ml at pH 7 [42]**.** In this study, chitinase activity declined significantly when the pH was increased or decreased indicating the significant effect of lower or higher pH. The results of this study, corroborated with the results reported in the literature *Paenibacillus* sp. [43,44]**,** *Bacillus* sp. [44]**,** *Brevibacillus laterosporus* strain PAP0 [45] chitinase produced by respectively at neutral pH.

### 3.7 Effects of incubation period on chitinase production

**Fig. 6:**
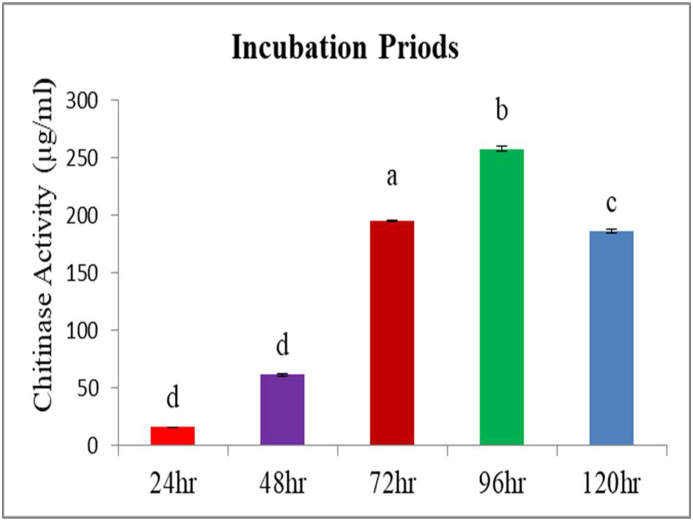
Effects of different incubation periods on the production of chitinase enzyme.

The evaluated different incubation periods significantly affected the chitinase enzyme activity (F_3_ = 485.9; P < 0.0001). The slowest enzyme activity was measured at 24 h incubation time for all bacterial strains and ranged from 8 μg/mL to 18 g μg/mL. The highest enzyme activity was observed at 96hr for the bacterial isolate *Stenotrophomonas maltophilia* (TSI1) 335 μg/mL followed by *Ligilactobacillus salivarius* HSIII1 265 μg/mL (Fig. 6). In this study, all remaining incubation times and the different bacterial isolates evaluated gave intermediate results between the highest and the lowest enzyme activities. The results of the incubation period in this study corroborates with the results reported in the literature. *Serratia marcescens* DSM 30121 T [46], *Cohnella* sp. A01, [47](*Streptomyces rubiginosus,* [48] *Penicillium chrysogenum,* [49] *Serratia* sp. and *Pseudomonas* sp*,* [50]. The results of this study revealed that the activity of the enzymes increased as incubation time increased up to 4th day and decreased then after.

## 4 Conclusions

In this work, chitinase-producing bacteria were isolated from soil samples collected from riverbanks, characterized, and phonetically 29 distinct isolates were obtained. Of these, 9 elite chitinolytic bacterial strains were chosen for further characterization and optimization experiments. By using a MALDI-TOF MS biotyper, nine out of the ten bacterial strains that were examined were identified at the species level. The optimal production of Chitinase was assessed based on a variety of different criteria The bacterial species *Stenotrophomonas maltophilia* (TSI1) and *Pseudomonas putida* TSIII1 both of which were isolated from Taltle river bank soils were best chitinase producers. at a temperature of 45 °C, neutral pH, and incubation temperatures of the 96hr. Thus, finding new chitinases appropriate for novel industrial applications could be made easier by screening microorganisms with higher activity levels. Purification and characterization of chitinase enzymes from the elite bacterial isolates for a wide range of application could be the direction of future research.

## CRediT authorship contribution statement

**Teshome Gudeta Gonfa:** Data curation, Formal analysis, Methodology, Software, Validation, Writing – original draft, Writing – review & editing, Conceptualization. **Asefa Keneni Negessa:** Supervision, Validation, Visualization. **Abebe Olani Bulto:** Resources, Investigation.

## Declaration of competing interest

The authors declare that they have no known competing financial interests or personal relationships that could have appeared to influence the work reported in this paper.

## Data Availability

The data that support the findings of this study are available on request from the corresponding author, [initials]. The data are not publicly available due to [restrictions e.g. their containing information that could compromise the privacy of research participants
